# Present Spatial Diversity Patterns of *Theobroma cacao* L. in the Neotropics Reflect Genetic Differentiation in Pleistocene Refugia Followed by Human-Influenced Dispersal

**DOI:** 10.1371/journal.pone.0047676

**Published:** 2012-10-24

**Authors:** Evert Thomas, Maarten van Zonneveld, Judy Loo, Toby Hodgkin, Gea Galluzzi, Jacob van Etten

**Affiliations:** 1 Regional Office for the Americas, Bioversity International, Cali, Colombia; 2 Faculty of Bioscience Engineering, Ghent University, Gent, Belgium; 3 Headquarters, Bioversity International, Rome, Italy; University College London, United Kingdom

## Abstract

Cacao (*Theobroma cacao* L.) is indigenous to the Amazon basin, but is generally believed to have been domesticated in Mesoamerica for the production of chocolate beverage. However, cacao’s distribution of genetic diversity in South America is also likely to reflect pre-Columbian human influences that were superimposed on natural processes of genetic differentiation. Here we present the results of a spatial analysis of the intra-specific diversity of cacao in Latin America, drawing on a dataset of 939 cacao trees genotypically characterized by means of 96 SSR markers. To assess continental diversity patterns we performed grid-based calculations of allelic richness, Shannon diversity and Nei gene diversity, and distinguished different spatially coherent genetic groups by means of cluster analysis. The highest levels of genetic diversity were observed in the Upper Amazon areas from southern Peru to the Ecuadorian Amazon and the border areas between Colombia, Peru and Brazil. On the assumption that the last glaciation (22,000–13,000 BP) had the greatest pre-human impact on the current distribution and diversity of cacao, we modeled the species’ Pleistocene niche suitability and overlaid this with present-day diversity maps. The results suggest that cacao was already widely distributed in the Western Amazon before the onset of glaciation. During glaciations, cacao populations were likely to have been restricted to several refugia where they probably underwent genetic differentiation, resulting in a number of genetic clusters which are representative for, or closest related to, the original wild cacao populations. The analyses also suggested that genetic differentiation and geographical distribution of a number of other clusters seem to have been significantly affected by processes of human management and accompanying genetic bottlenecks. We discuss the implications of these results for future germplasm collection and *in situ,* on farm and *ex situ* conservation of cacao.

## Introduction

Numerous studies have been dedicated to investigating the genetic diversity of cacao (*Theobroma cacao* L.) [1]–[8], but most of these had a local or at most national scope. The first larger scale investigation, covering representative sites of the current distribution pattern of cacao in the whole of Latin America was recently published by Motamayor et al. [9]. The latter authors genotyped 1241 trees, leading them to propose a new classification of the currently known cacao germplasm. Here we use part of this dataset to investigate spatial diversity patterns of cacao at the continental level. Available information about spatial patterns and gene flow in cacao is still scarce [5] and the work that does exist relates to patterns at relatively small scale. For example, Zhang et al. [5], [7] found significant spatial correlations at regional scale in Peru and Bolivia, respectively, providing evidence for the hypothesis of isolation by distance in cacao populations. This long-expected pattern has been related to the limited, short-distance gene flow in cacao, and the fact that self-pollination may be more common than assumed in natural populations [3], [4], [7], [10].

A better understanding of the spatial distribution of genetic diversity in cacao is important because it can contribute to improving our knowledge of the temporal and spatial dynamics of this economically important crop [5], which in turn underlie the species’ adaptability to environmental change [11]. Furthermore, it can provide information to guide the identification of priority areas for (i) collection of promising germplasm material for *ex situ* conservation and potential use in breeding programs, and (ii) *in situ* conservation (cf. [12]). Finally, geospatial diversity analyses can help confirm or refute cacao’s putative center of origin, and improve our understanding about possible historical dispersal routes [13].

Genetic differentiation triggered by local adaptation of geographically separated (sub)populations of a species is in many cases the result of evolutionary processes running over hundreds to thousands of generations. Current intraspecific diversity patterns of many Amazonian plant species are at least in part a reflection of their distribution during the last period of glaciation (22,000–13,000 BP), which had the greatest impact on the vegetation of northern South America in Pleistocenic history [4]. There seems to be growing consensus that during the Last Glacial Maximum (LGM∼21,000 BP) the Amazon Basin experienced significant cooling, combined with a reduction in precipitation, and water stress in plants due to lowered atmospheric CO_2_ concentrations [14]–[18]. As a consequence of this, part of the Amazon Basin, or at least the ecotonal areas towards its northern and southern margins, was probably occupied by non-rainforest vegetation, such as (more open) dry forest, and in some areas even savannah [14], [16], [17], [19], [20]. Consequently, the floristic composition and structure of the vegetation in the Amazon basin at the LGM were probably also quite different from what they are today [15], [16], [18]. The most notable example is possibly the occurrence of typical Andean taxa like *Podocarpus* or *Alnus* in the Amazon basin [14], [15], [18], but it is likely that climatic conditions during the LGM also affected the Amazonian distribution of cacao, e.g. by restricting it to a number of geographically and genetically isolated refugia as suggested by Motamayor et al. [1].

During the glacial-Holocene transition, evergreen rainforest distribution probably increased again owing to ameliorating climatic and CO_2_ conditions [18]. During the Early-Mid-Holocene (ca. 8000–3600 years ago) there was a new drop in precipitation causing seasonal widespread, frequent fires in southern Amazonia, and increased abundance of dry forest taxa and savannahs in ecotonal areas. Finally, in the Late Holocene rainforests expanded once more because of increased precipitation [18]. Considering that different responses to climate change may be expected from different species, depending on their adaptation and environmental tolerance [16], [21], here we model the potential past distribution of cacao based on the average of two climate models of the LGM by means of Ecological Niche Modeling. This approach has been used successfully for reconstructing past potential distributions of species, e.g. to better understand their current distribution and diversity [17], [22].

For species with longstanding economic or livelihood importance like cacao and Brazil nut, varying levels of past human intervention contributed to shaping current spatial diversity patterns [23]. In the case of cacao, human-mediated dispersal probably began in the warming period in the Holocene after the initial peopling of the Amazon which started around 11,200 BP at the latest [24]. It is clear now that cacao was brought to Mesoamerica by early humans where use and domestication of this crop may have started some 4,000 years ago [25], but humans also played an important role in distributing the species over the Amazon Basin [24]. It has been hypothesized that certain cacao populations, particularly those from the lower Amazon, might derive from selection and domestication processes conducted by pre-Colombian Amazonian peoples for the aromatic pulp of the fruits [3], [5], [24], [26]–[29]. As is the case for several other *Theobroma* and *Herrania* species, the pulp surrounding cacao seeds was a popular snack and was fermented to make an alcoholic beverage, and occasionally vinegar, by numerous Amazonian indigenous groups at the time of European contact [27], a practice which persists until today [30]. In fact, preparation of alcoholic beverages from cacao pulp may have led to the discovery of the usefulness of cacao seeds for preparing chocolate in Central America [25], [29].

In addition, when considering recent findings concerning the biological characteristics of cacao in wild populations (rare flowers, pollination by small midges with limited action-sphere, short pollination distances, general self-incompatibility, indehiscent fruits, recalcitrant seeds, seed dispersal over short distances, and a high degree of vegetative reproduction in wild populations), and the aggregated occurrence of cacao trees in natural stands [3], [4], [7], [10], it seems implausible that the current continent-wide distribution of cacao was entirely due to natural processes. Although sporadic seed dispersal under natural conditions has been reported to be mediated by monkeys, birds, squirrels and even deer [27], this does not seem to have been very efficient [4], possibly because its original megafauna dispersal agent(s) went extinct [31].

The present paper has three objectives. First we investigate the distribution of genetic diversity of cacao in Latin America (alpha diversity) and identify areas holding the highest levels of genetic diversity under the assumption that these may correspond with areas where original wild populations can be found, or at least populations that are most related to the original wild populations (cf. [1]). Wild populations that are adapted to locally prevailing environmental conditions are of interest because they can contain genetic material for potential use in breeding programs, e.g. for improving disease or drought resistance. Our second objective is to describe and explain the spatial distribution of different genetic groups or clusters that can be distinguished in the cacao dataset here analyzed (beta diversity). Hereby we explicitly take into consideration the historical distribution of cacao, as well as the impact of historical human activities. Finally, we interpret the results obtained in terms of their implication for future germplasm collection and *in situ,* on farm and *ex situ* conservation of cacao.

## Methods

### Datasets

A number of different data sources were analysed to produce the results presented in this paper. Most importantly, we used the open-access dataset elaborated by the Agricultural Research Service (ARS) of the United States Department of Agriculture (USDA), which contains microsatellite marker data from 1241 cacao individuals, evaluated over 106 loci (available at http://www.ars.usda.gov/Research/docs.htm?docid=16432). This same dataset formed the basis for the paper of Motamayor et al [9] who cleaned the data by eliminating mislabeled accessions, duplicates, hybrids, and markers yielding inconsistent observations. As such, the authors retained data for 952 individuals and 96 microsatellite markers. This germplasm originates from 12 countries and was collected between 1937 and 2005 (for further information see [9]). The latter dataset formed the starting point for the present paper. We removed two points from Ecuador for which coordinates were missing and two points from Ghana. Subsequently, we performed additional cleaning with respect to consistency between administrative units mentioned in the passport data, and observed after projection of the geographic coordinates, and applied the reverse jackknife method integrated in DIVA-GIS [32] to identify climatic outliers. The latter analysis was based on the bioclimatic values associated with all of the cacao records, extracted from 2.5 minute rasters of the 19 bioclim variables, obtained from the Worldclim website [33]. This data cleaning exercise resulted in the exclusion of 9 additional South American data points. Hence the microsatellite dataset used in the present paper consisted of 939 cacao individuals evaluated with 96 markers.

For the distribution modeling of cacao under past, current, and future climatic conditions, we extracted additional georeferenced observation points from GBIF (www.gbif.org) to obtain a more representative distribution of cacao growing sites in Latin America. After the previously described data cleaning, a total of 1333 cacao records were retained (i.e. the 939 records with genetic data and 394 additional ones). We performed the same exercise for obtaining representative observation points for cacao’s wild relatives (i.e. the 19 other species in the *Theobroma* genus (APG [34] and The Plant List [35] website, accessed October 2011)), leading to 1636 additional (non-cacao) records.

### Genetic Parameters

The neutral SRR genetic marker data used in the current study are particularly useful for investigating processes such as gene flow, migration or dispersal [36]. Neutral molecular marker diversity can additionally provide useful indications about the level of historic and/or ongoing genetic isolation of populations, and hence to a certain extent also about their potential for adaptation to local environmental conditions, as a consequence of such isolation. Of particular interest in this respect are what Frankel et al. [37] called locally common alleles. These are alleles -or DNA sequences- that only occur in a limited part of the total area of a species, but in those areas have a relatively high frequency, which, in combination, are indicative for the level of genetic isolation and possibly also local adaptation.

In this paper, genetic parameters were calculated following two different approaches: one based on grid cells and the other based on genetic clusters. Grid-based calculations of genetic parameters included allelic richness per locus, the number of locally common alleles per locus (i.e. alleles occurring in 25% or less of all grid cells and with a frequency of at least 5% in a grid cell), Shannon information index and Nei gene diversity [38]. Cluster-based calculations of genetic parameters included the average number of private alleles per locus, i.e. alleles that only occur in one single cluster, and average observed heterozygosity in addition to the ones previously listed. Locally common alleles for clusters were calculated as the average number of alleles per locus occurring in one or two clusters and with a frequency higher than 5% in a cluster.

### Grid-based Spatial Diversity Analyses

#### Circular neighborhood

Grid-based spatial diversity analyses were performed using 10 minute grid cells (∼18 km at the equator), constructed in ESRI ArcMap 10, as the unit of analyses. To obtain sufficient and more evenly distributed data points for constructing high resolution maps of genetic diversity at a continental scale, we applied circular neighborhoods of one degree diameter around the locations of all the cacao trees considered in this paper, following van Zonneveld et al. [12]. With this we assume that each of the sampled trees is representative for the circular area with one decimal degree diameter (∼111 km at the equator) around it. Consequently, each tree was replicated in all the 10 minute grid cells contained in a one degree diameter around its location. This replication exercise resulted in a total dataset of 26,067 trees, distributed over 1,678 grid cells of 10 minutes size (note that the original dataset consisted of 939 trees (i.e. the trees with microsatellite data considered in this paper) distributed over 138 grid cells). For a more in-depth discussion of the circular neighborhood methodology, please refer to [12].

#### Bootstrap correction of sample bias

The previously described circular neighborhood technique does not in itself eliminate the sampling bias that was already present in the original dataset: the number of trees per grid cell after the replication exercise varied between 1 and 244. To allow for standardized and statistically sound comparisons of genetic parameters between grid cells containing different numbers of trees, these parameters were calculated on bootstrapped subsamples (without repetition) of trees per grid cell. The bootstrap approach generates highly similar results as the rarefaction methodology that is commonly used for correcting the sample bias in allelic richness calculations [39], but has the advantage that it can be used for correcting sample bias of any genetic parameter and not just allelic richness. We set the sample size equal to the median of the distribution of the number of trees per grid cell, i.e. 8 trees or 16 gene copies (g = 16) (see [40] for terminology). By applying this threshold value (i.e. discarding all cells containing less than 8 trees), we retained 55% and 90% of the 1,678 grid cells and 26,067 trees obtained after the replication exercise. For each of the retained grid cells, we averaged the values obtained for each of the genetic parameters as calculated for 1,000 bootstrap samples. Calculations were performed in R statistical package version 2.14 [41]. The R script of this bootstrap sample bias correction methodology is freely available from the authors on request. To assess whether 1,000 bootstrap repetitions were sufficient for obtaining an acceptable level of precision, we applied the rarefaction algorithm in ADZE program [42] on the cacao dataset using a sample size of 16 genecopies (g = 16,) and compared the values obtained with the results from our respective bootstrap calculations. This resulted in values of 0.99 and r = 0.99 for regression slope coefficient and Pearson correlation coefficient, respectively, justifying the validity of the methodology used.

### Spatial Principal Components Analysis

To visualize continental-scale gradients in the genetic diversity of cacao and enhance our understanding of how geographical and environmental features structure this genetic diversity, we applied a spatial principal components analysis (sPCA) [43] in adegenet package version 1.3–2 [44] for the statistical program R. This method yields scores that summarize both the genetic variability and spatial structure among individuals. It uses both a matrix with allele frequencies of genotypes and a spatial weighting matrix containing measurements of spatial proximity among entities, based on a connection network [43]. Here we used the Delaunay triangulation algorithm for constructing the connection network between the sampled cacao trees. More specifically, sPCA uses Moran’s I to measure spatial structure in allele frequency values of samples. Moran’s I values are highly positive when allele frequency values observed at neighboring sites tend to be similar (contributing to global structures in data), whereas it is strongly negative when allele frequency values at neighboring sites tend to be dissimilar (contributing to local structures). An sPCA generates two sets of axes: one set with positive eigenvalues and the other with negative eigenvalues. Positive eigenvalues correspond to global structures, while negative values are indicative of local patterns. Applied to the present cacao dataset, a very strong global structure was detected. The positive eigenvalues of the first two axes were clearly much higher than all other eigenvalues and therefore we only interpreted the first global structure associated with the first two axes. This decision was confirmed by a Monte-Carlo test on the global and local structures in the dataset (simulated p-values <0.001 and 0.78, respectively). We visualized the global structure in the cacao dataset on a raster map with 10 minute grid cells, by assigning to each cell the average value of the projections on the first sPCA axis of all individuals enclosed by a circular neighborhood of one degree diameter constructed around its center.

### Identification and Characterization of Genetic Clusters

We used adegenet to identify different genetic groups in the cacao dataset. To identify the optimal number of clusters this package runs the *k*-means algorithm with increasing values of *k* on the PCA-transformed data (i.e. the 939 genotyped trees) and then compares different clustering solutions using Bayesian Information Criterion (BIC). It is important to bear in mind that in many (most) cases the ultimate choice of the optimal *k* value is user-defined. In this respect Jombart [45] says that “clustering algorithms help make a mere caricature of a complex reality which is most of the time far from following known population genetics models”. There simply is no true *k*, but some values of *k* are better, more efficient summaries than others [45]. For the present analysis we chose the same number of ten clusters as used by Motamayor et al [9] which was backed up by the first noticeable elbow in the BIC curve generated by adegenet. Membership probabilities of each individual cacao tree for the different groups were calculated in adegenet by means of a discriminant analysis of principal components (DAPC) on the previously determined clusters. These membership values are different from the admixture coefficients of software like STUCTURE (used by Motamayor et al [9]) but can be interpreted in a similar fashion as proximities of individuals to the different clusters [45]. Ninety-eight percent of the 939 individual trees had cluster membership values larger than 0.7 (more than half of the remaining trees had membership values over 0.6). Separate raster maps were constructed for each of the clusters with cells of 10 minutes. For this, only individuals with membership values of at least 0.7 were considered. Each cell was assigned the highest membership value of the individuals enclosed by a circular neighborhood of one degree diameter constructed around its center. To visualize the genetic similarity of clusters we additionally constructed a dendrogram in R package vegan 1.17–12 [46] based on Nei’s distance and using the complete linkage clustering algorithm. This yielded a cophenetic correlation value of 0.92 confirming the validity of using this method. To compare genetic parameters among the different clusters of different sizes (varying between 35 and 158 individuals with membership values ≥0.7), we applied the bootstrapping method described above, using a sample size of 35 (the size of the smallest cluster) and 1,000 repetitions.

To compare the climatological niches occupied by the different clusters, we extracted the values of the 19 bioclim variables and altitude for each of the sampled trees corresponding to their location from the respective 2.5 minute raster maps. Furthermore, to allow visualizing the environmental niches of the different clusters in two-dimensional space, we applied a Principal Coordinates Analysis (PCoA) by means of R packages Vegan 1.17–12 and BiodiversityR 1.5 [47], using Nei’s distance as a distance parameter. A posteriori, we performed vector fitting in Vegan to visualize the importance of the different bioclim variables and altitude, as well as the correlations between them. As input for this ordination exercise we used (1) an allele matrix with the cacao individuals in rows, the SSR loci in columns and corresponding alleles in cells, and (2) an environmental matrix with the cacao individuals in rows, the 19 bioclim variables and altitude in columns and the corresponding values in cells. All the environmental variables were significant (p = 0.001; permutation tests). Highly similar results were obtained from a distance-based redundancy analysis (i.e. the constrained analog of PCoA). However, given that cacao trees -even abandoned cultivars- are able to survive naturally in appropriate humid forest ecosystems [24], and given that a large part of cacao’s current distribution is due to human dispersal processes (cf. discussion), we do not believe it is appropriate to characterize the ecological niches in ordinate space where the axes are constricted to linear combinations of the environmental variables here considered.

### Niche Modeling

We characterized the spatial distribution of suitable habitat conditions of cacao under current, past, and future climatic conditions, by means of the ecological niche modeling algorithm implemented by Maxent version 3.3.3e [48], using the default settings. Maxent identifies potential distribution areas on the basis of their similarity in environmental conditions, compared to those at the sites where the species has already been observed. We trained the model based on the extended dataset of 1,333 cacao records (see above) and the current monthly climate data at 2.5′ spatial resolution obtained from the Worldclim database (i.e. averages from 1960–1990 [33]; www.worldclim.org/current) and projected it on past and future climate scenarios at the same resolution. The Area Under Curve value obtained was 0.912, pointing to good model performance. For past climate conditions we used the average of the two downscaled climate models of the Last Glacial Maximum (LGM; ∼ 21000 yr BP) obtained from the WorldClim database (www.worldclim.org/past). It is highly probable that processes of expansion and constriction of species distributions occurred also in times preceding the LGM [16], but we assume that current spatial patterns in the intraspecific diversity of cacao most strongly reflect the impacts of climate change during the LGM [4]. For characterizing future climate conditions, we averaged 19 downscaled climate models for 2050 based on the A2 scenario [49] of greenhouse gas emissions (International Center for Tropical Agriculture; available at http://ccafs-climate.org/). We restricted the modeled distributions visualized on maps to the maximal threshold value generated by Maxent (maximum training sensitivity plus specificity (cf. [50]), here 0.37 for the logit threshold) to compensate for the uncertainty associated with the (averages of the) past and future climate models used.

For developing a map of the modeled species richness of genus *Theobroma*, we ran Maxent’s ecological niche modeling algorithm for each of the species for which a reasonable number of georeferenced observations were available in GBIF, using the current monthly climate data at 2.5′ spatial resolution obtained from the WorldClim database. Five species for which only between 1 and 3 records were available were excluded from this analysis. All records of these species are located in South America, three of which in western Amazonia. Numbers of records for the retained *Theobroma* species (excluding cacao) varied between 8 and 670, and values obtained for the Area Under Curve were higher than 0.9 (except for one species for which a value of 0.84 was obtained), suggesting good model performance in all cases. Next we made binary (presence-absence) maps of each of the species at the same resolution, applying Maxent’s ten percentile training presence threshold values and overlaid these rasters so as to obtain a map showing for each cell the number of *Theobroma* species with modeled distribution in that cell. A map of the observed species richness of genus *Theobroma* (i.e. showing the distribution of records extracted from GBIF) was developed in DIVA-GIS at resolution of 10 minutes and a circular neighborhood of one degree.

## Results

### Alpha Diversity Patterns

**Figure 1:**
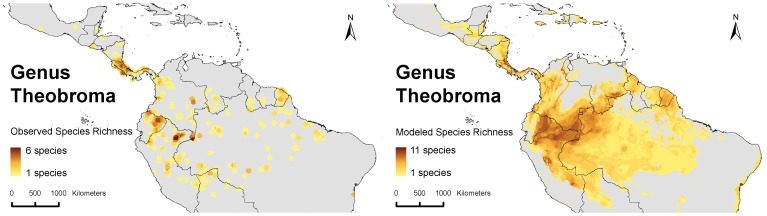
Species richness of genus *Theobroma*. Left: observed species richness in 10 minute grid cells and a circular neighborhood of 1 decimal degree; Right: modeled species richness in 2.5 minute grid cells.

Highest species richness of genus *Theobroma* is observed in southern Nicaragua, Costa Rica and western Panama (observed and modeled species richness up to 5 and 11, respectively) on the one hand and the Upper Amazon regions of Ecuador, Northern Peru and southern Colombia (observed and modeled species richness up to 6 and 9, respectively), as well as northeastern Brazil and southern Venezuela (figure 1).

**Figure 2:**
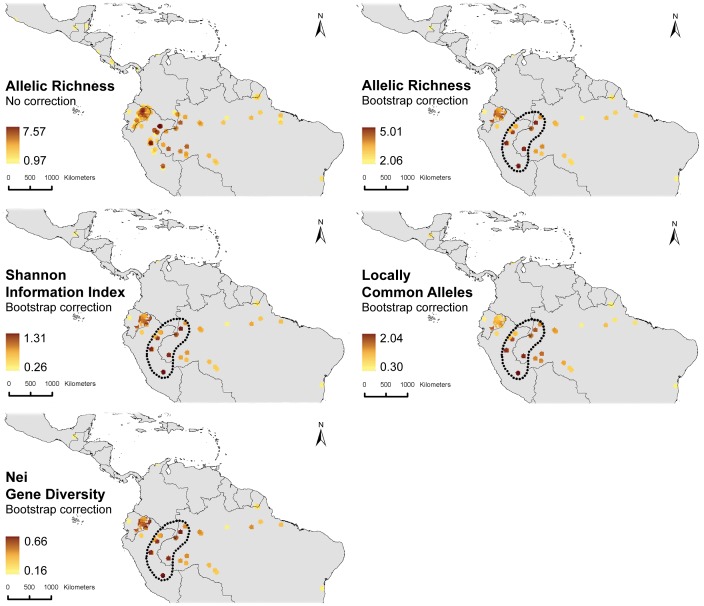
Spatial variation of different genetic parameters, represented at a resolution of ten minute grid cells and a circular neighborhood of 1 degree. Highest values are consistently observed in the extensive bean-shaped Amazonian area covering both the Peruvian-Brazilian border, and the southern part of the Colombian-Brazilian border, as well as Amazonian Ecuador.

An overview of the spatial distribution of several genetic diversity parameters calculated for *Theobroma cacao,* to which the rest of our analyses are dedicated, is given in Figure 2. First of all, a comparison of allelic diversity with (right) and without (left) bootstrap correction shows different patterns in the distribution of the genetic diversity of cacao, confirming the importance of correcting sample bias in the dataset. Second, the different genetic parameters show a fairly consistent pattern whereby the highest values are observed in the extensive bean-shaped Amazonian area covering both the Peruvian-Brazilian border, and the southern part of the Colombian-Brazilian border. Comparably high values are also observed in Amazonian Ecuador. It is notable that the highest values were consistently found in central (i.e. the southern part of Loreto Department) and south-Eastern Peru (i.e. the Amazonian area of Cuzco department).

**Figure 3:**
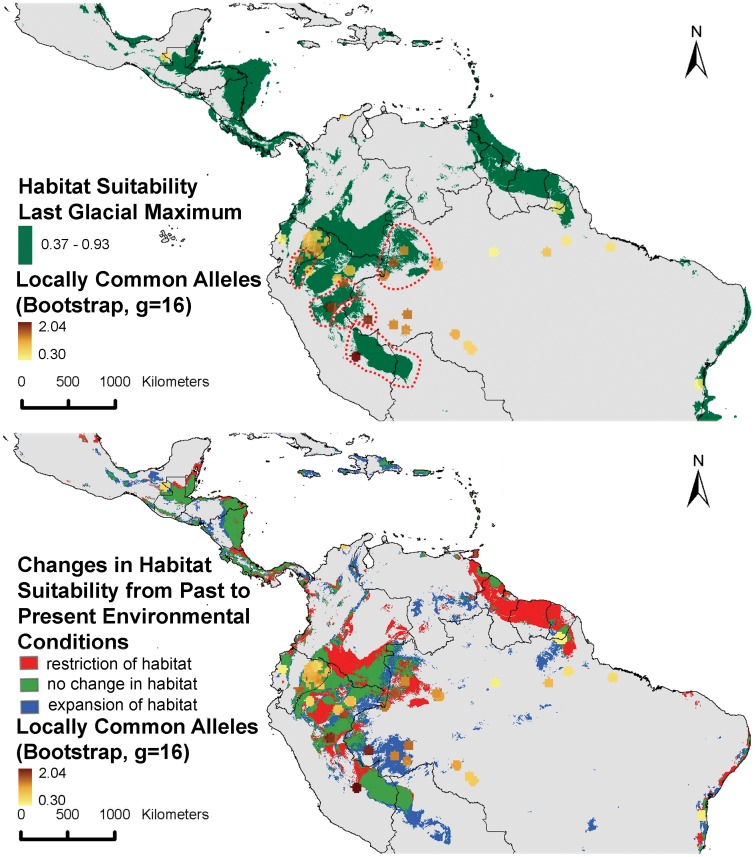
Observed locally common alleles compared to past and current modeled distribution of cacao. Upper: distribution of areas with modeled habitat suitability of cacao during the LGM; red dashed polygons show potential relatively isolated refugia associated with areas holding high levels of locally common alleles. Lower: changes in cacao habitat suitability from the LGM until present; red areas represent potential habitat suitability during LGM but no longer at present (high impact or restriction areas); green indicates areas with continued habitat suitability from LGM until present (low impact or stable areas); and blue indicates areas that were probably not suitable for cacao at the LGM, but are suitable at present (new or expansion areas).

We applied environmental niche modeling to investigate whether the current patterns observed in cacao alpha diversity can be related to its potential distribution during the last glacial maximum (LGM; ∼21,000 years BP). Figure 3 shows that the Amazonian areas where suitable habitat conditions for cacao prevailed during the LGM may have been quite extensive, but at the same time also quite fragmented whereby some of these fragments could have acted as isolated refugia. While it is highly unlikely that cacao was already present in the LGM refugia areas of Central America or the Brazilian east coast, it is less straightforward to rule out potential cacao survival in the extensive refugium area covering the Guyana shield due to the lack of genetic observations from that area.

In any case, only in the western Amazon, a certain level of correspondence seems to exist between the areas where the highest levels of locally common alleles (and other genetic parameters) were observed and some potential refugia (figure 3). As argued above, high values of locally common alleles may be indicative of local adaptation of cacao populations to environmental conditions prevailing in isolated refugia. The red dashed polygons in figure 3 are only intended to indicate potential approximate areas in which genetic differentiation may have taken place, based on available data, and should not be considered as rigid interpretations of LGM refugia. Figure 3 additionally shows that some of the sampling areas where highest locally common allele values were observed correspond to areas to which cacao populations expanded from the potential LGM refugia (indicated by the fact that they are located in blue areas). This expansion seems to have been mainly directed towards the center of the Amazon basin.

### Beta Diversity

**Figure 4:**
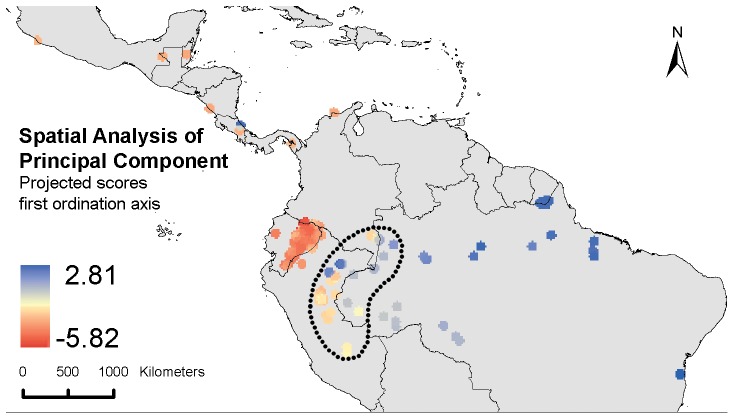
Scores of sampled trees as projected on the first ordination axis of the biplot of a Spatial Analysis of Principal Components. Location of the genetic cline coincides with the bean-shaped area (and particularly the southern part of it) where the highest values of the measured genetic parameters were observed (figure 2).

A Mantel test comparison of the geographical distances with Nei’s genetic distance between individual cacao trees showed strong evidence for isolation by distance (r = 0.27; p<0.001), which was expected given the continental scale of the dataset. A better visual representation of continental-scale gradients in the genetic diversity of cacao is obtained through projection of the results of a spatial Analysis of Principal Components (sPCA) on a map of the study area (figure 4). Interestingly, the location of the genetic cline identified by the first global scores (scores on the first sPCA axis) coincides with the bean-shaped area (and particularly the southern part of it) where the highest values of the measured genetic parameters were observed (figure 2). Furthermore, it tends to suggest that the Central American cacao populations are more related with their Ecuadorean counterparts and less so with those from the Colombian, Peruvian and Brazilian Amazon.

**Figure 5:**
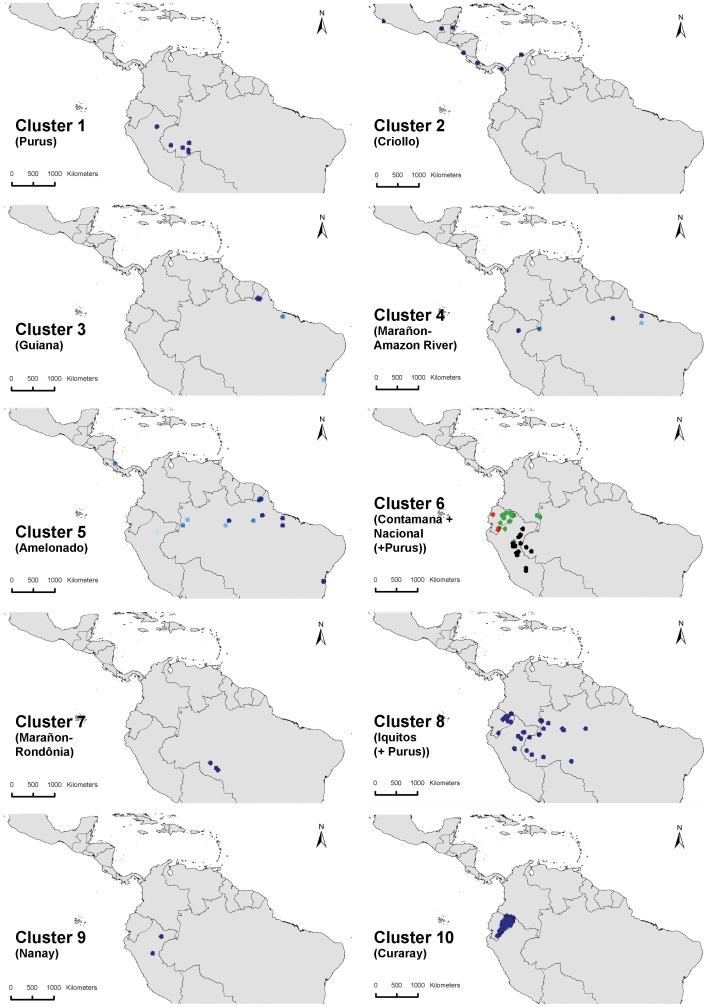
Overview of the different locations of the ten clusters identified by k-means clustering. The three subclusters of cluster 6 are highlighted with different colours, clearly distinguishing the group that is largely composed of the Nacional cultivar of the Ecuadorean coastal plains (red colour).

The 10 clusters obtained from k-means clustering in adegenet (figure 5) correspond quite well with the clusters described by Motomayor et al [9]. Half of our clusters made a perfect match, i.e. Criollo (our cluster2), Guiana (cluster 3), Amelonado (cluster 5), Nanay (cluster 9) and Curaray (cluster 10). By contrast, in the present analysis the Marañon cluster of [9] was split into two different clusters (one associated with the Amazon River (cluster 4)) and one located in Rondônia (cluster 7)), whereas the Contamana and the Nacional were joined in a single cluster (cluster 6). Most individuals assigned to the Purus cluster by [9] were also assigned to one cluster in our analysis (cluster 1), but the entire “Upper Solimões R[iver] Iça R[iver]” subcluster was grouped together with the individuals of the Iquitos cluster (cluster 8). A number of other individuals of the Purus cluster for which [9] generally found low membership values and hence did not assign them to any subcluster, in our analysis were partly assigned to clusters 6 and 8. It is important to stress that the purpose of this clustering is clearly not to challenge the genetic groups described by [9]. We merely use these groups to try to uncover some of the general patterns underlying the observed spatial distribution of cacao’s genetic diversity.

Somewhat surprisingly, and as opposed to the result obtained by [9], the Nacional cultivar from coastal Ecuador was not assigned to a separate cluster by the k-means algorithm applied here (while the Criollo and Amelonado cultivars did cluster consistently). In fact, all 20 cacao trees in the dataset from the Ecuadorian coastal area obtained membership values of 100% for cluster 6, which is hence the only cluster that occurs in this area. However, when repeating k-means clustering for k = 3 in adegenet only for individuals with membership values of at least 0.7 within cluster 6, the Nacional cluster as identified by [9] could be separated (figure 5). Most of the trees (20) in this subcluster are located on the coastal plains of Ecuador, but also two trees that are located at the Amazonian side of the Andes were included (red colored dots). This suggests that the germplasm that lead to the development of the Nacional cultivar crossed the Andes in southern Ecuador [9] where the mountains are lower, hence facilitating human movements.

**Figure 6:**
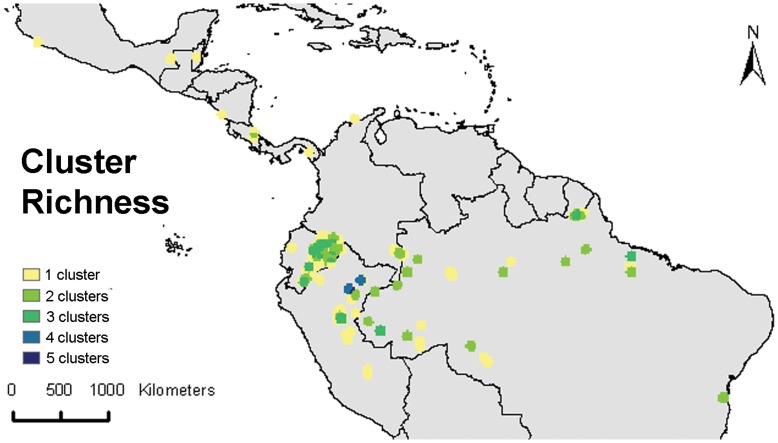
Cluster richness, i.e. the number of different clusters shown in **figure 5** that occur in a given area.

Highest cluster diversity is observed in northeastern Peru, in the region around and to the south of Iquitos, with up to 4–5 different clusters occurring in this relatively small area (figure 6). Up to three clusters are observed in areas from the Ecuadorean Amazon and the Brazilian states of Acre and Para, respectively. High cluster richness around Iquitos is partly due to sample bias whereby many more collections were made in this region as compared to other areas.

Large variation exists in the measured genetic parameters from one cluster to another (table 1). Clusters 6, 8, 1 and 10 show the highest levels of genetic diversity for nearly all parameters measured (mean allelic richness values per locus without bootstrap correction for these clusters were as high as 11.05, 10.23, 6.73 and 6.89, respectively). It is remarkable that a relatively high value was calculated for locally common alleles for the least diverse and highly homozygous Criollo cultivar (cluster 2). A closer look at the twenty cacao trees from coastal Ecuador, corresponding to the Nacional cultivar, in comparison with other trees from cluster 6 reveals the enormous difference between the two groups in terms of genetic diversity (table 2). Furthermore it shows that the genetic diversity parameters obtained for the Nacional cultivar are highly comparable to those of the Criollo cultivar. The so called “pure Nacional”, same as the “pure Criollo”, has white beans (D. Zhang, personal communication).

**Table 1:** Averages of genetic parameters per locus for clusters 1 to 10, based on 1,000 bootstrap samples of 35 trees (i.e. the size of the smallest cluster).

	Allelic Richness	Shannon Information Index	Locally Common Alleles	Private alleles	Nei Gene Diversity	Observed Heterozygocity
**Cluster 6** (Contamana+ Nacional (+Purus))	8.02	1.50	0.28	0.70	0.68	0.40
**Cluster 8** (Iquitos(+ Purus))	7.12	1.33	0.13	0.17	0.63	0.55
**Cluster 1** (Purus)	5.75	1.04	0.17	0.26	0.51	0.40
**Cluster 10** (Curaray)	5.23	1.00	0.18	0.16	0.50	0.36
**Cluster 4** (Marañon – Amazon river)	4.23	0.88	0.04	0.07	0.47	0.45
**Cluster 5** (Amelonado)	3.67	0.63	0.04	0.02	0.33	0.15
**Cluster 9** (Nanay)	3.66	0.56	0.03	0.04	0.29	0.25
**Cluster 7** (Marañon – Rondônia)	3.33	0.66	0.12	0.07	0.36	0.30
**Cluster 3** (Guiana)	2.41	0.40	0.03	0.01	0.23	0.11
**Cluster 2** (Criollo)	1.77	0.18	0.17	0.10	0.10	0.02

Four highest values for each of the parameters are underlined.

**Table 2:** Averages of genetic parameters per locus for trees from coastal Ecuador (Nacional cultivar) and the remaining trees from cluster 6 (Contamana + Nacional (+Purus)), based on 1,000 bootstrap samples of 20 trees (i.e. the number of trees from coastal Ecuador).

	Allelic Richness	Shannon Information Index	Private alleles	Nei Gene Diversity	Observed Heterozygocity
**Nacional cultivar**	1.53	0.21	0.05	0.13	0.12
**Other trees**	7.14	1.50	5.53	0.69	0.44

**Figure 7:**
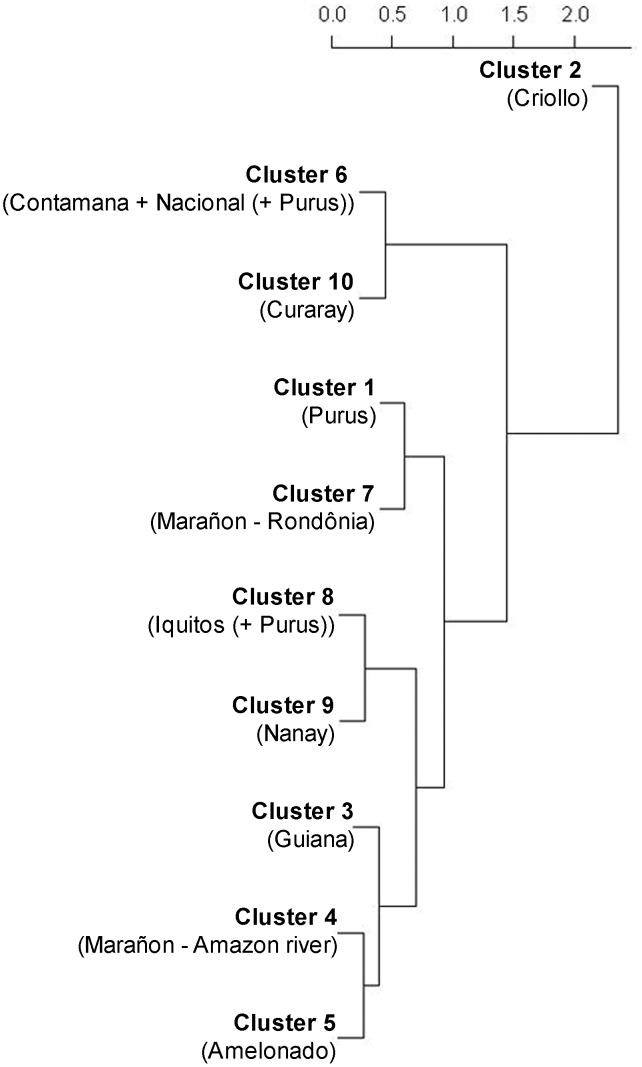
Complete linkage clustering based on Nei’s distance (cophenetic correlation = 0.92).

Figure 7 shows a dendrogram of the different clusters described above. Cluster 2 (Criollo cultivar) is genetically most separated from all other clusters (average Nei distance 1.82) and shows most affinity with cluster 6 (1.32). When comparing the genetic distances between the Criollo group and the subclusters of cluster 6 identified in figure 5, the Nacional group was more dissimilar (1.82) than the other two subclusters (1.39 and 1.45 for the green and black subclusters in figure 5 respectively).

The remaining clusters are grouped together more or less in accordance with the geographical range they occupy. Cluster 6 and 10 are separated by a relatively small genetic distance (0.44) and both occur closest to the Andean foothills (figure 5). Cluster 8 has the lowest mean distance compared to all other clusters (0.64 on average) and shows relatively high similarity with clusters 9 (Nei distance 0.28), 5 (0.34) and 4 (0.37). The smallest distance was observed between clusters 4 and 5 (0.27) and both these are proximate to cluster 3 (0.32 and 0.39, respectively). This could suggest that these clusters differentiated genetically from a common gene pool. Geographically, these clusters also occupy the area from central to eastern Amazonia (figure 5). Finally, cluster 1 and 7 are grouped together (Nei distance 0.61), which is in line with the fact that they are geographical neighbors (figure 5).

**Figure 8:**
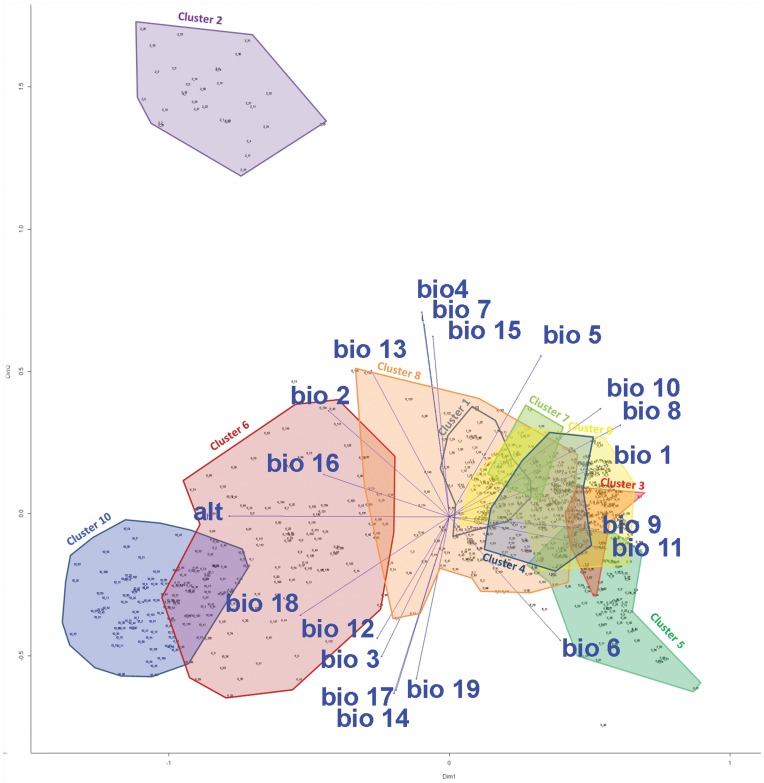
Ordination diagram of a Principal Coordinate Analysis applied on the cacao dataset, using Nei’s distance. The first two axes show 69% of the variation in data. Environmental variables were added *a posteriori* through vector fitting. Arrows point in the direction of most rapid change in the variable and their length is proportional to the correlation between ordination and variable. According to the classification used by [9], Cluster1 = Purus; Cluster2 = Criollo; Cluster3 = Guiana; Cluster4 = Marañon-Amazon River; Cluster5 = Amelonado; Cluster6 = Contamana + Nacional (+Purus); Cluster7 = Marañon-Rondônia; Cluster8 = Iquitos (+Purus); Cluster9 = Nanay; Cluster10 = Curaray (alt = altitude; BIO1 = Annual mean temperature; BIO2 = Mean diurnal range (max temp – min temp) (monthly average); BIO3 = Isothermality (BIO1/BIO7) * 100; BIO4 = Temperature Seasonality (Coefficient of Variation); BIO5 = Max Temperature of Warmest Period; BIO6 = Min Temperature of Coldest Period; BIO7 = Temperature Annual Range (BIO5–BIO6); BIO8 = Mean Temperature of Wettest Quarter; BIO9 = Mean Temperature of Driest Quarter; BIO10 = Mean Temperature of Warmest Quarter; BIO11 = Mean Temperature of Coldest Quarter; BIO12 = Annual Precipitation; BIO13 = Precipitation of Wettest Period; BIO14 = Precipitation of Driest Period; BIO15 = Precipitation Seasonality (Coefficient of Variation); BIO16 = Precipitation of Wettest Quarter; BIO17 = Precipitation of Driest Quarter; BIO18 = Precipitation of Warmest Quarter; BIO19 = Precipitation of Coldest Quarter).

The different clusters occupy different ecological niches (p-values<0.0001; Kruskall-Wallis tests for all variables here considered). A Mantel test confirmed the relation between genetic distance (Nei) and environmental distance (Euclidean) (r = 0.38, p = 0.001). The PCoA ordination diagram in figure 8 gives an approximate representation of how the realized niches of the different clusters relate to one another. The pattern observed corresponds relatively well with the outcome of the clustering exercise depicted in figure 7. Cluster 2 occupies an ecological niche that is quite different from all other clusters. It grows in areas with much higher temperature and precipitation seasonality. However, bearing in mind that Criollo cacao was introduced to Central America by humans, it is unlikely that this extreme ecological niche is entirely due to adaptation to local environmental conditions. Individuals of clusters 10 and -to lesser extent- cluster 6 occur at higher altitudes and higher levels of precipitation during the warmest and wettest quarters of the year than the other South American clusters. Cluster 8 occupies an intermediate ecological niche between clusters 6 and 10 on the one hand, and the remaining clusters on the other hand, which occupy much more similar niches. This is in line with the previous observation that cluster 8 has the lowest mean distance compared to all other clusters.

**Figure 9:**
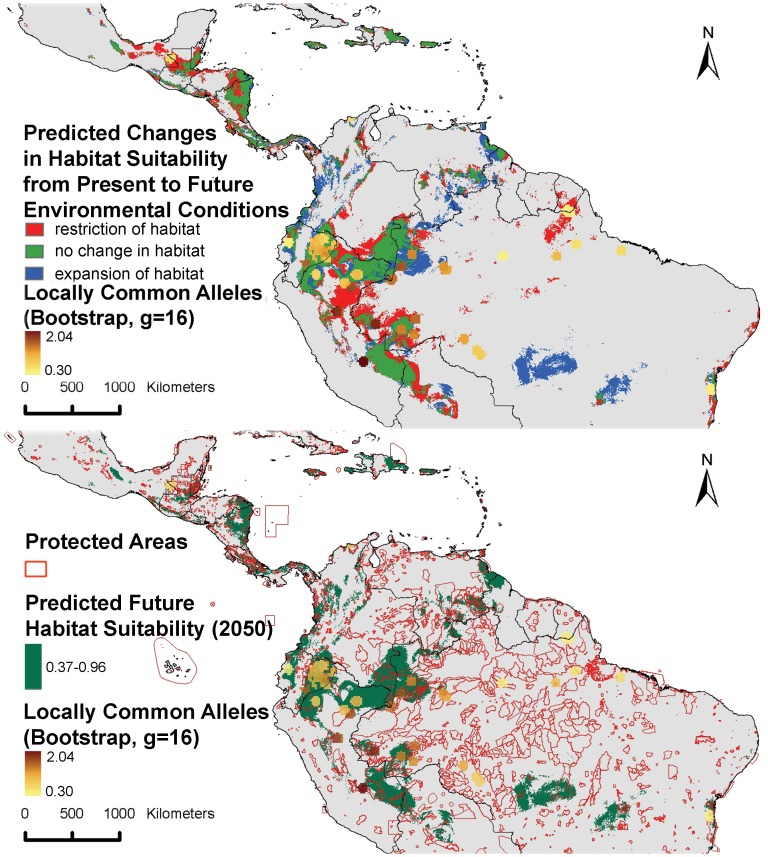
Observed locally common alleles compared to current and future modeled distribution of cacao. Upper: predicted changes in cacao habitat suitability from present until 2050; red areas represent potential habitat suitability at present but no longer by 2050 (high impact or restriction areas); green indicates areas with continued habitat suitability from present until 2050 (low impact or stable areas); and blue indicates areas which are currently unsuitable for cacao, but may become suitable by 2050 (new or expansion areas) Lower: distribution of areas with modeled habitat suitability of cacao by 2050, overlaid with the location of currently existing protected areas.

To assess the *in situ* conservation status of cacao in light of future climate change, we modeled its potential distribution based on the average of 19 currently available downscaled climate models for 2050 and the A2 greenhouse gas emission scenario. Although we do not expect this model to give a 100% trustworthy high resolution map of the 2050 niche suitability of cacao, through the use of a high threshold value (see methodology) it should give us a fair approximation of the main areas where the species will have a high likelihood of surviving. Figure 9 shows that environmental change will likely cause considerable shifts in the current distribution range of the species. Particularly in the extensive bean-shaped Amazonian area covering both the Peruvian-Brazilian border and the southern part of the Colombian-Brazilian border, which we previously identified as the region of highest genetic diversity (figure 2), a considerable negative impact is expected with a net decrease in habitat suitability. However, a closer look (lowermost map of figure 9) shows that cacao should be able to survive in the vicinities of all locations where the highest values of the genetic parameters here considered were recorded. Significant parts of these suitable habitats for cacao are located in protected areas which should, in principle, safeguard these areas and allow for in situ survival and conservation of cacao populations. The region around Iquitos in northeastern Peru, where both high levels of genetic diversity (figure 2) and the highest number of clusters (figure 6) were observed, is the most notable area where the potential future distribution of cacao is not significantly secured in protected areas.

## Discussion

### Role of Historical Factors in Contemporary Spatial Diversity Patterns of Cacao

The regions with highest diversity in *Theobroma* species are the upstream areas of the western Amazon, and to a lesser extent the southern part of Central America (figure 1). However, it is likely that most, if not all, *Theobroma* species are not native to Central America [1], [27], [29], [51]. This would suggest that the origin of the *Theobroma* genus may be situated in the Western Upper Amazon region which is in line with Bartley’s [52] argument that the Peruvian Amazon is a center of diversity for the genus *Theobroma*. The fact that there exists a certain level of overlap between the areas of highest species richness of the *Theobroma* genus and the area where the highest genetic diversity of cacao is observed corroborates the hypothesis that cacao also originated in the Western Upper Amazon.

Interpretation of the Pleistocene modeled distribution of cacao together with present-day genetic observations suggests that the species was already distributed widely across the Western Amazon prior to the LGM. Furthermore, the model suggests that the glaciation cooling and drying may have led to restriction of cacao’s distribution to a number of relatively isolated refugia centers (figure 3) where precipitation remained fairly high, within a broader Amazonian mosaic composed of different vegetation types. Several of these putative refugia are located at the foot of the Andes where humid forests seem to have prevailed during the late Pleistocene [14]–[16]. It has been shown before that intraspecific diversification occurred in the Middle-Late Pleistocene [53]–[55]. In line with this, and as suggested by Motamayor et al [1], it is likely that long-term constrained gene flow in LGM refugia led to genetic differentiation between isolated cacao populations prior to the subsequent phase of forest expansion in the warming period of the Holocene, including to the areas where some of the observations of the dataset here considered were made.

The locations of most putative refugia (figure 3) correspond to a certain extent with the locations where the genetic parameters considered here, and particularly locally common alleles, attain their maximum values (figure 2). However, the paleo-distribution model of cacao also predicts a high probability of favorable habitat conditions for cacao in extensive areas covering the northern Peruvian and southern Colombian Amazon for which no representative observation points were included in the present dataset. More data from these areas are necessary for a more comprehensive understanding of the extent and distribution of potential LGM refugia of cacao.

The current distribution of cacao and of most, if not all, of the clusters described previously (figure 5) are likely to have been partly shaped by a varying degree of human intervention in the warming period in the Holocene, superimposed on ongoing natural processes of range contraction and expansion. The consistently lower values of genetic parameters observed along the margins of the cacao distribution area -likely to be the consequence of human-induced bottlenecks-, and the fact that the most important cacao cultivars occur along the margins corroborate this hypothesis. Likewise, the observation of high values for genetic parameters (figure 2), and cluster richness in the area around Iquitos-Peru (figure 6) may be partly the consequence of human intervention whereby material from different areas was brought to the Iquitos region (cf. [9]) and possibly further selection and cultivation of cacao took place for its edible pulp [24]. This is in line with the fact that the Iquitos region was an important center of crop genetic resources at the time of European conquest [24], [28] and that major pre-conquest population centers concentrated crop genetic resources to guarantee their subsistence and survival [28]. Also the fact that the pulp of several other species in the *Theobroma* genus was used in a similar fashion as cacao by South-American indigenous groups [27] might explain why the present-day highest species richness of genus *Theobroma* is located in this very same area (figure 1).

In sum, we concur with other authors [1], [3], [5] that the center of genetic diversity of cacao is located in the upper Amazonian regions bordering Peru, Brazil, Colombia and Ecuador. Our observations are less conclusive about a putative origin of cacao in the Upper Amazon near the Colombian-Ecuadorian border [56]. In this reasoning we made the following assumptions: (i) cacao populations were already more widely distributed prior to the LGM, at least in Western Amazonia, complicating the tracing back of the true center of origin; (ii) these populations were restricted to a number of relatively isolated refugia during the period(s) of cooling and drying in the Pleistocene; and (iii) they expanded again during the Holocene warming, partly due to natural range expansion and partly due to human-mediated dispersal. Van Etten and Hijmans [13] recently proposed a method for tracing back the putative origin of a crop based on the assumption that one would expect a regular decline in observed heterozygocity of a crop when moving away from its origin. The highest levels of observed heterozygocity of individual trees in the current dataset were consistently observed in the vicinities of the putative refugia here identified (results not shown). Application of the latter authors’ model-based approach could possibly shed more light on the validity and patterns of post-GLM spread of cacao from these refugia.

### Spatial Distribution and Genetic Differentiation of Cacao Clusters

The previously described general spatial diversity patterns are reflected in the geographical distribution and diversity of the ten clusters described above. Based on the values of the genetic parameters obtained for the different clusters (table 1), the clusters that are most likely closest related to original wild populations are cluster 6 (Nacional + Contamana (+Purus) clusters of [9]), cluster 8 (Iquitos (+Purus)), followed by cluster 10 (Curaray) and cluster 1 (Purus). Mean allelic richness per locus in these clusters is comparable to the allelic richness observed for the upper Amazon Forastero group by Motamayor and Lanaud ([26]; 8.69 alleles per locus – but based on smaller set of markers), who assume that these high values are indicative for wild populations, or at least populations that are most closely related to the original wild populations. The present results also corroborate previous findings that the highest number of private alleles are typically observed in the Upper Amazon region of Peru and Brazil [3], [9].

The origin of cluster 6 (Nacional + Contamana (+Purus)) seems to be located in the southern part of Amazonian Peru since it is the only prevailing cluster (figures 5 and 6) and highest values are observed for genetic parameters (figure 2). Zhang et al [7] also found high levels of cacao genetic diversity (average allelic richness of 8.93 and gene diversity of 0.74 per locus) close to the region in Peru where cluster 6 possibly originated (Ucayali cacao germplasm). The fact that this cluster extends to the Ecuadorian Pacific coast suggests that material from southern Peru could have been the original genepool that led to the Nacional cultivar. If this is true, cacao germplasm from the original southern-Peruvian genepool that differentiated in local refugia (figure 3) could have been moved northwards by pre-Columbian people, and eventually crossed the Andes [24], [57]. As suggested by Loor et al [2], the size of the ancestral population of the Nacional that was brought to coastal Ecuador was very small, leading to a profound bottleneck and low diversity values.

The center of origin of cluster 8 (Iquitos (+Purus)) may well be located in the upper Amazon region of northwestern Brazil, close to the corresponding refugium (figure 3). As for cluster 6 this is based on the observation that this is one of the few areas where the highest levels of genetic parameters have been observed in the present study (figure 2) and cluster 8 is either the only cluster found here, or in combination with cluster 5 (Amelonado; figures 5 and 6), which may have evolved from the same original genepool as cluster 8 (fourth smallest Nei distance between clusters 5 and 8).

Cluster 10 (Curaray) is restricted to Amazonian Ecuador and may have developed from a part of the original cacao population that was isolated during the LGM and resulted in relatively high values of genetic parameters observed in some parts of this region (figure 2). The fact that none of the trees from this cluster occur outside Amazonian Ecuador (not even with low membership values) (figure 5), points to a local differentiation of this cluster. The observation that the previous three clusters are the only ones found in Ecuador (and the Ecuadorian-Colombian border), and that clusters 6 and 8 probably originated in different areas in Peru and Brazil, respectively, would argue against an Ecuadorian origin of cacao as a species. In addition, the fact that clusters 6 and 8 both occur in the Ecuadorian and Peruvian Amazon, while cluster 10 is restricted to the Ecuadorian Amazon suggests that material was only distributed upwards, from Peru to Ecuador, and not vice versa.

The remaining cluster that is notable in terms of observed values for genetic parameters is cluster 1 (Purus) whose potential origin is located in the Brazilian state of Acre (figure 5). This cluster is also associated with a refugium center (figure 3) and prevails in an area where the highest levels of genetic parameters were observed per grid cell. Furthermore, the high genetic diversity is paralleled by high phenotypic diversity for various morphological, molecular and agronomic characters observed in the region [3].

The clusters that follow the previous ones in terms of high values registered in the genetic parameters are clusters 4 (Marañon-Amazon River) and 5 (Amelonado). It is likely that the genetic differentiation of these clusters was triggered by human interference [3], [24]. Both these clusters also occur in the Iquitos area (remember: a center of crop genetic resources diversity) and were probably spread to the eastern part of the Amazon by early humans [24]. The fact that, at the time of contact, there were centers of crop genetic resources in the lower Amazon [28] corroborates this assumption. These centers are the Central Amazonian Center, that may have extended along the main stream of the Amazon River, from the Purus River in the west to the Tapajós River in the east, and included the lower sections of the Negro, Madeira and Tapajós Rivers, and the Marajó -Island Minor Center, in the region of the mouth of the Amazon river in the Atlantic Ocean [28]. According to Clement [28] cacao was already available in these centers at the time of contact. Hence we tend to agree with Sereno et al [3] that part of the cacao populations in the lower Amazon region are probably derived from natural stands from the upper Amazon region which underwent genetic bottlenecks associated with continued selection by humans, which would explain their lower levels of allelic richness and high degree of inbreeding. As such it is probable that cluster 5 (Amelonado), and possibly also cluster 4 (Marañon-Amazon River), originated from the same genepool that led to the genetic differentiation of cluster 8 (Iquitos (+Purus)). Given that the lowest genetic distance observed between clusters in the dataset was precisely between clusters 4 and 5 and that clusters are synthetic simplifications of reality, it is perfectly possible that under another clustering scenario individuals from both clusters would be grouped together.

Cluster 9 (Nanay) is a small cluster located in the northern part of the Peruvian Amazon (figure 5). It seems to be strongly associated with cluster 8 (Iquitos (+Purus); figure 7) and both may have differentiated genetically from a common gene pool. Motamayor et al [9] found that individuals of both clusters were grouped together when redoing the clustering exercise based on a subsample of 15 individuals from each of the 10 clusters. This again illustrates that clusters are artificial representations of reality and should be interpreted with caution. At the same time, the fact that cluster 9 obtained from the present analysis made a perfect match with Motamaoyor et al’s [9] Nanay cluster may be indicative for a certain level of genetic differentiation (possibly human-mediated, given its location close to Clement’s [28] center of crop diversity located around present-day’s Iquitos).

Cluster 7 (Marañon-Rondônia) is located in Rondônia and is geographically and genetically associated with cluster 1 (Purus; figure 7). Almeida and Almeida [58] observed that cacao populations from this region were morphologically homogenous and suggested that these populations should form part of a large complex of cacaos trees which are genetically related. Sereno et al [3] found comparably low levels of allelic richness (3.0 alleles per locus) and observed heterozygosity (0.288) as for cluster 7 in the present research. It is interesting to note that cluster 7 is located in an area outside the modeled distribution of cacao (figure 9) which may suggest that individuals from this cluster actually represent cultivated trees.

Cluster 3 (Guianas) mainly occurs in eastern South America (figure 5). The relatively low values for the measured genetic parameters (the second lowest of all clusters), and the fact that there is a weak signal of this cluster in the Iquitos area, suggest that also this cluster differentiated genetically through human-induced genetic bottlenecks during the Holocene [24], in a similar fashion as clusters 4 (Marañon-Amazon River) and 5 (Amelonado). However, on the basis of the present data (figure 3), it cannot be ruled out that this cluster may represent a relic of cacao populations that were already available in the Guianas prior to the LGM, and survived in local refugia during the period of cooling and drying, as suggested by [4]. The latter authors obtained comparable levels of allelic richness (2.40), observed and expected heterozygosity (0.157 and 0.285, respectively) as the mean values we calculated for cluster 3.

Cluster 2 corresponds to the Criollo cultivar. This cluster shows the lowest levels of genetic diversity in the dataset, most likely resulting from genetic bottlenecks that accompanied human selection and domestication processes [1]. The present data are inconclusive to determine whether cacao arrived to Central America either from coastal Ecuador through dispersal routes along the pacific coast, or rather through northern migration routes along the continental side of the Andes. Based on the observation that according to the clustering scheme proposed by [9] the Criollo cultivar is also found in northwestern Ecuador (observation not taken into account in the present analysis because identified as climatic outlier, cf. methodology), Clement *et al*. [24] expressed support for the first hypothesis. By contrast, Motamayor and Lanaud [26] argued -in support of the second hypothesis- that historic and genetic evidence point to south-western Venezuela as a potential area where domestication of the Criollo group may have started. Also the fact that today only Criollo trees are found in the latter area would corroborate this hypothesis. In line with this, the Criollo cacao in Ecuador may have been introduced from Venezuela, because historically there was an intensive introduction of cacao from Venezuela to northeastern Ecuador (D. Zhang, personal communications).

Based on the highest genetic affinity of cluster 2 with cluster 6 (Nacional + Contamana (+Purus)) found in the present analysis, it seems that the Criollo cultivar may be derived from an original southern Peruvian genepool (from which cluster 6 may also have originated). The Criollo cluster shows also higher affinity with the northernmost subcluster of cluster 6 (figure 5) of which representatives are found in southeastern Colombia, relatively close to southwestern Venezuela, than with the Nacional subcluster of cluster 6 of coastal Ecuador, providing support for the second hypothesis mentioned higher. However, it cannot be ruled out that cacao germplasm crossed the Andes and consequently was moved northwards up to Central-America, before the start of human selection in coastal Ecuador that led to the Nacional cultivar. Vice versa, it is perfectly possible that the Criollo trees presently found in northwestern Ecuador or southwestern Venezuela are southward reintroductions from Central-America, after conclusion of the genetic differentiation of the Criollo cultivar there. Future application of modeling approaches such as those proposed by van Etten and Hijmans [13] could possibly be used to either confirm current hypotheses or to identify alternative solutions.

### Implications for Germplasm Collection and Conservation

Most of the main clusters described above occupy different ecological niches. This could imply that trees from a particular niche (particularly trees growing at the high or low end of a niche; e.g. with exceptionally low or irregularly distributed precipitation) may contain alleles that give them competitive advantage to survive in that niche, compared to trees from sites with different environmental conditions. Such information can be useful when planning collection trips in search of interesting traits for potential use in cacao breeding programs.

The likelihood of encountering interesting genetic material for breeding is higher where levels of (neutral) genetic diversity attain their maximal values simply because the high neutral diversity implies that populations did not experience genetic bottlenecks [12], [59]. Particularly, high levels of locally common alleles are interesting because they can indicate isolation of populations, which in turn may foster natural selection and local adaptation of genotypes if environmental conditions differ from other areas. For example, genetic material from Upper Amazon cacao trees (which typically show highest diversity levels) has often been used in breeding programs due to their strength, precocity and resistance to disease [5], [6], [60]. Based on the previous, and taking into account the probable constriction of cacao in refugia during the LGM and posterior range extension, priority areas for germplasm collection missions would be areas where (1) high levels of genetic diversity and locally common alleles are observed; and (2) cacao has been able to survive since the LGM, because the genetic diversity in expansion areas may not attain the same levels as in areas where local adaptation possibly took place. Hence *in concreto*, interesting areas for collection of cacao germplasm would be the green areas in figure 3 that are situated in the vicinities of observations of high levels of genetic diversity and locally common alleles, such as the Peruvian Madre de Dios department. It is interesting to note that most of the locations where high genetic diversity and locally common alleles were observed are situated at the margins or in expansion areas of the LGM refugia. This might raise chances of finding additional interesting genetic material more towards the centers of the potential refugia.

However, some nuancing is in place here, because the neutral genetic variation measured here is not generally linked with genetic variation and population differentiation at quantitative, adaptive traits [11], [36]. More research is needed to evaluate the usefulness of neutral markers in cacao for identifying populations with potentially interesting adaptive traits for breeding programs. For example, the fact that cacao trees show low neutral genetic diversity does not necessarily mean that they cannot contain interesting functional or adaptive traits [11]. The best examples are the Nacional and Criollo cultivars for which the lowest genetic diversity was recorded. This is partly the consequence of (1) human-induced bottlenecks, whereby the size of the genepool that led to the development of these cultivars was strongly reduced, and partly due to (2) selection and domestication processes, whereby the frequency of favorable traits was culturally enhanced, which in turn led to the best quality fine-flavor chocolate known today. This probably also explains the relatively elevated number of locally common alleles observed for cluster 2 (Criollo; table 1). Hence, in spite of their highly homozygous nature and higher susceptibility to pests and diseases [2], [60], these cultivars contain important organoleptic traits of human interest. It is perfectly possible that other trees with relatively low diversity (eg the recently characterized Bolivian Nacional [7] or the aromatic *Chuncho* variety from Cuzco) also contain interesting functional traits.

In any case, it is important to stress that prior to planning new collection missions, it is essential to obtain a full understanding of what is currently conserved in existing *ex situ* collections. This has not yet been done and is important to avoid duplication. Furthermore, *ex situ* conservation needs to be complemented by *in situ* and on farm conservation of diverse wild and cultivated populations, respectively [61]. Such populations are exposed to evolutionary stress and/or human-mediated selection, which allows for continued adaptation to a changing environment [11], and higher frequencies of desirable traits, respectively. Our preliminary analysis of the mid-term (2050) conservation status of cacao suggests that, in spite of possible drastic range contractions, most of cacao’s genetic capital should be able to withstand near-future climatic changes and survive in protected areas. The biggest gap in the protection of interesting and highly diverse cacao populations may be situated in northeastern Peru, in the Amazonian region around Iquitos. Not only were high levels of overall genetic diversity observed here (figure 2), but it is also the area hosting the highest number of different genetic clusters (figure 6). In addition, our niche model predicts a high likelihood for cacao populations to survive here in the future (figure 9). This is an important detail because the true value of protected areas lies in their ability to sustain target plant population for perpetuity [14]. The availability of a wide variety of building blocks in this genetic melting pot may provide evolution with the necessary elements to respond more flexibly to future environmental change. Therefore, this may be a priority area for conservation of cacao, preferably through a combination of (a network of) protected areas and on-farm conservation.

### Future Challenges

This study has shown the merit of using spatial diversity analysis to uncover patterns in genetic marker data that may remain ‘hidden’ when using more classical approaches to population genetics. The approach adopted has allowed us to begin to separate the possible contributions of climate change, geography, history and culture to the current distribution of genetic diversity in cacao. Such modeling procedures may be applicable to other crops as well. Although the extensive dataset used in this paper allowed a better understanding of the spatial distribution of genetic diversity in cacao, several questions remain unanswered. Further validation the hypotheses we have put forward here can probably best be achieved through a combined application of alternative modeling approaches (e.g. [13], [62]) and groundtruthing, e.g. through future collections at strategically chosen sites, or by verifying the model ‘fit’ of already available (genetic) observations (from other studies or *ex situ* collections). Standardized use of molecular markers is crucial in this respect [12]. More specifically, more data from the Colombian and northern Peruvian Amazon could improve our knowledge about the extent and distribution of potential Pleistocene refugia, whereas additional data from Venezuela could allow us to verify the status of the supposedly wild populations reported by early chroniclers from the area of the Orinoco river [27] and to assess their relation with the lower Amazon Amelonado type [1]. Another interesting area to include in future would be Bolivia (Cacao Nacional Boliviano) where the southwestern limit of natural distribution of cacao is situated [7]. Observed gene diversity in this area is lower than in southern Peru [7], but it would be interesting to investigate if Bolivian cacao also originates from Peruvian stock or represents an isolated group that is derived from cacao germplasm that differentiated genetically in a southern refugium, as suggested by [7].

## References

[1] Motamayor JC, Risterucci AM, Lopez PA, Ortiz CF, Moreno A, et al (2002) Cacao domestication I: the origin of the cacao cultivated by the Mayas. Heredity 89: 380–386. 12399997 10.1038/sj.hdy.6800156

[2] Loor RG, Risterucci AM, Courtois B, Fouet O, Jeanneau M, et al (2009) Tracing the native ancestors of the modern *Theobroma cacao* L. population in Ecuador. Tree Genetics & Genomes 5: 421–433.

[3] Sereno ML, Albuquerque PSB, Vencovsky R, Figueira A (2006) Genetic diversity and natural population structure of cacao (*Theobroma cacao* L.) from the Brazilian Amazon evaluated by microsatellite Markers. Conservation Genetics 7: 13–24.

[4] Lachenaud P, Zhang D (2008) Genetic diversity and population structure in wild stands of cacao trees (*Theobroma cacao* L.) in French Guiana. Annals of Forest Science 65: 310.

[5] Zhang D, Arevalo-Gardini E, Mischke S, Zúñiga-Cernades L, Barreto-Chavez A, et al (2006) Genetic diversity and structure of managed and semi-natural populations of cocoa (*Theobroma cacao*) in the Huallaga and Ucayali Valleys of Peru. Annals of botany 98: 647–655. 16845139 10.1093/aob/mcl146 PMC3292056

[6] Zhang D, Boccara M, Motilal L, Mischke S, Johnson ES, et al (2009) Molecular characterization of an earliest cacao (*Theobroma cacao* L.) collection from Upper Amazon using microsatellite DNA markers. Tree Genetics & Genomes 5: 595–607.

[7] Zhang D, Martínez WJ, Johnson ES, Somarriba E, Phillips-Mora W, et al (2012) Genetic diversity and spatial structure in a new distinct *Theobroma cacao* L. population in Bolivia. Genetic Resources and Crop Evolution 59: 239–252.

[8] Trognitz B, Scheldeman X, Hansel-Hohl K, Kuant A, Grebe H, et al (2011) Genetic population structure of cacao plantings within a young production area in Nicaragua. PloS ONE 6: e16056. 21264251 10.1371/journal.pone.0016056 PMC3021531

[9] Motamayor JC, Lachenaud P, da Silva E Mota JW, Loor R, Kuhn DN, et al (2008) Geographic and genetic population differentiation of the Amazonian chocolate tree (*Theobroma cacao* L). PloS ONE 3: e3311. 18827930 10.1371/journal.pone.0003311 PMC2551746

[10] Silva CRS, Albuquerque PSB, Ervedosa FR, Mota JWS, Figueira A, et al (2011) Understanding the genetic diversity, spatial genetic structure and mating system at the hierarchical levels of fruits and individuals of a continuous *Theobroma cacao* population from the Brazilian Amazon. Heredity 106: 973–985. 21139632 10.1038/hdy.2010.145 PMC3186253

[11] Pautasso M (2009) Geographical genetics and the conservation of forest trees. Perspectives in Plant Ecology, Evolution and Systematics 11: 157–189.

[12] van Zonneveld M, Scheldeman X, Escribano P, Viruel MA, Van Damme P, et al (2012) Mapping genetic diversity of cherimoya (*Annona cherimola* Mill.): application of spatial analysis for conservation and use of plant genetic resources. PLoS ONE 7: e29845. 22253801 10.1371/journal.pone.0029845 PMC3253804

[13] van Etten J, Hijmans RJ (2010) A geospatial modelling approach integrating archaeobotany and genetics to trace the origin and dispersal of domesticated plants. PLoS ONE 5: e12060. 20711460 10.1371/journal.pone.0012060 PMC2920323

[14] Bush MB (1996) Amazonian conservation in a changing world. Biological Conservation 76: 219–228.

[15] Bush MB, Oliveira PED (2006) The rise and fall of the refugial hypothesis of Amazonian speciation: a paleoecological perspective. Biota Neotropica 6.

[16] Haffer J, Prance GT (2001) Climatic forcing of evolution in Amazonia during the Cenozoic: on the refuge theory of biotic differentiation. Amazoniana 16: 579–607.

[17] Bonaccorso E, Koch I, Peterson AT (2006) Pleistocene fragmentation of Amazon species’ ranges. Diversity & Distributions 12: 157–164.

[18] Mayle FE, Beerling DJ, Gosling WD, Bush MB (2004) Responses of Amazonian ecosystems to climatic and atmospheric carbon dioxide changes since the last glacial maximum. Philosophical Transactions of the Royal Society of London Series B, Biological sciences 359: 499–514. 15212099 10.1098/rstb.2003.1434 PMC1693334

[19] Beerling DJ, Mayle FE (2006) Contrasting effects of climate and CO 2 on Amazonian ecosystems since the last glacial maximum. Global Change Biology 12: 1977–1984.

[20] Pennington RT, Lavin M, Prado DE, Pendry CA, Pell SK, et al (2004) Historical climate change and speciation: Neotropical seasonally dry forest plants show patterns of both Tertiary and Quaternary diversification. Philosophical Transactions of the Royal Society of London Series B: Biological Sciences 359: 359–537. 10.1098/rstb.2003.1435 PMC1693336 15212100

[21] Stewart JR, Lister AM, Barnes I, Dalén L (2010) Refugia revisited: individualistic responses of species in space and time. Proceedings of The Royal Society Biological sciences 277: 661–671. 19864280 10.1098/rspb.2009.1272 PMC2842738

[22] Waltari E, Hijmans RJ, Peterson AT, Nyári AS, Perkins SL, et al (2007) Locating pleistocene refugia: comparing phylogeographic and ecological niche model predictions. PloS ONE 2: e563. 17622339 10.1371/journal.pone.0000563 PMC1905943

[23] Shepard G, Ramirez H (2011) “Made in Brazil”: human dispersal of the Brazil Nut (*Bertholletia excelsa*, Lecythidaceae) in ancient Amazonia. Economic Botany 65: 44–65.

[24] Clement CR, de Cristo-Araújo M, d’ Eeckenbrugge GC, Alves Pereira A, Picanço-Rodrigues D (2010) Origin and Domestication of Native Amazonian Crops. Diversity 2: 72–106.

[25] Powis TG, Cyphers A, Gaikwad NW, Grivetti L, Cheong K (2011) Cacao use and the San Lorenzo Olmec. Proceedings of the National Academy of Sciences of the United States of America 108: 8595–8600. 21555564 10.1073/pnas.1100620108 PMC3102397

[26] Motamayor, JC; Lanaud C (2002) Molecular analysis of the origin and domestication of *Theobroma cacao* L. In: Engels, JMM, Ramanatha Rao, V, Brown, AHD, Jackson MT, editors. Managing Plant Genetic Diversity. Rome. 77–87.

[27] Patiño Rodriguez VM (2002) Historia y dispersión de los frutales nativos del Neotropico. Cali, Colombia: Centro Internacional de Agricultura Tropical.

[28] Clement CR (1999) 1492 and the loss of Amazonian crop genetic resources. II. Crop biogeography at contact. Economic Botany 53: 203–216.

[29] Henderson JS, Joyce RA, Hall GR, Hurst WJ, McGovern PE (2007) Chemical and archaeological evidence for the earliest cacao beverages. Proceedings of the National Academy of Sciences of the United States of America 104: 18937–18940. 18024588 10.1073/pnas.0708815104 PMC2141886

[30] Thomas E (2012) The impact of traditional lifestyle, provenance and contact history on plant knowledge: a comparison of two small-scale societies from the Bolivian Amazon. Human Ecology 40: 355–368.

[31] Guimaraes PR, Galetti M, Jordano P (2008) Seed dispersal anachronisms: rethinking the fruits extinct megafauna ate. PLoS ONE 3: e1745. 18320062 10.1371/journal.pone.0001745 PMC2258420

[32] Hijmans RJ, Guarino L, Cruz M, Rojas E (2001) Computer tools for spatial analysis of plant genetic resources data: 1. DIVA-GIS. Plant Genetic Resources Newsletter 127: 15–19.

[33] Hijmans RJ, Cameron SE, Parra JL, Jones PG, Jarvis A (2005) Very high resolution interpolated climate surfaces for global land areas. International Journal of Climatology 25: 1965–1978.

[34] Stevens PF (2001 onwards) Angiosperm Phylogeny Website. Version 9, June 2008 [and more or less continuously updated since].

[35] The Plant List (2010) Version 1. Published on the Internet. Available:http://www.theplantlist.org.

[36] Holderegger R, Kamm U, Gugerli F (2006) Adaptive vs. neutral genetic diversity: implications for landscape genetics. Landscape Ecology: 797–807.

[37] Frankel OH, Brown AHD, Burdon J (1995a) The genetic diversity of wild plants. In: The conservation of plant biodiversity. Cambridge, Great Britain: University Press. 10–38.

[38] Nei M (1973) Analysis of Gene Diversity in Subdivided Populations. Proceedings of the National Academy of Sciences 70: 3321–3323. 10.1073/pnas.70.12.3321 PMC427228 4519626

[39] Leberg PL (2002) Estimating allelic richness: effects of sample size. Molecular Ecology 11: 2445–2449. 12406254 10.1046/j.1365-294x.2002.01612.x

[40] Comps B, Go D, Petit RJ (2001) Diverging trends between heterozygosity and allelic richness during postglacial colonization in the European Beech. Genetics 157: 389–397. 11139519 10.1093/genetics/157.1.389 PMC1461495

[41] R Development Core Team (2011) R: A language and environment for statistical computing. Version 2.14. R Foundation for Statistical Computing, Vienna, Austria.

[42] Szpiech Z, Jakobsson M, Rosenberg N (2008) ADZE: a rarefaction approach for counting alleles private to combinations of populations. Bioinformatics 24: 2498–2504. 18779233 10.1093/bioinformatics/btn478 PMC2732282

[43] Jombart T, Devillard S, Dufour A-B, Pontier D (2008a) Revealing cryptic spatial patterns in genetic variability by a new multivariate method. Heredity 101: 92–103. 18446182 10.1038/hdy.2008.34

[44] Jombart T (2008b) adegenet: a R package for the multivariate analysis of genetic markers. Bioinformatics 24: 1403–1405. 18397895 10.1093/bioinformatics/btn129

[45] Jombart T (2011) A tutorial for Discriminant Analysis of Principal Components (DAPC) using adegenet 1.3–0. vignette for R package.

[46] Oksanen J, Blanchet FG, Kindt R, Legendre P, O’Hara RB, et al.. (2011) vegan: Community Ecology Package. R package version 1.17–12.

[47] Kindt R, Coe R (2005) Tree diversity analysis. A manual and software for common statistical methods for ecological biodiversity studies. Nairobi, Kenya: World Agroforestry Centre (ICRAF).

[48] Phillips S, Anderson R, Schapire R (2006) Maximum entropy modeling of species geographic distributions. Ecological Modeling 190: 231–259.

[49] Sankovski A, Barbour W (2000) Pepper W (2000) Quantification of the IS99 emission scenario storylines using the atmospheric stabilization framework (ASF). Technological Forecasting & Social Change 63: 263–287.

[50] Liu C, Berry PM, Dawson TP, Pearson RG (2005) Selecting thresholds of occurrence in the prediction of species distributions. Ecography 28: 385–393.

[51] Dickau R (2005) Resource use, crop dispersals, and the transition to agriculture in prehistoric Panama: evidence from starch grains and macroremains. PhD dissertation, Temple University.

[52] Bartley GD (2005) The genetic diversity of cacao and its utilization. Wallingford, UK: CABI Publishing.

[53] d’ Horta FM, Cabanne GS, Meyer D, Miyaki CY (2011) The genetic effects of Late Quaternary climatic changes over a tropical latitudinal gradient: diversification of an Atlantic Forest passerine. Molecular Ecology 20: 1923–1935. 21410807 10.1111/j.1365-294X.2011.05063.x

[54] Resende HC, Yotoko KSC, Delabie JHC, Costa MA, Campiolo S, et al (2010) Pliocene and Pleistocene events shaping the genetic diversity within the central corridor of the Brazilian Atlantic Forest. Biological Journal of the Linnean Society 101: 949–960.

[55] Solomon SE, Bacci M, Martins J, Vinha GG, Mueller UG (2008) Paleodistributions and comparative molecular phylogeography of leafcutter ants (Atta spp.) provide new insight into the origins of Amazonian diversity. PloS ONE 3: e2738. 18648512 10.1371/journal.pone.0002738 PMC2447876

[56] Cheesman E (1944) Notes on the nomenclature, classification and possible relationships of cocoa populations. Tropical Agriculture 21: 144–159.

[57] Loor RG, Fouet O, Lemainque A, Pavek S, Risterucci A-M, et al.. (2012) Origin and domestication of the Nacional cacao variety from Ecuador and collecting expedition of new related genetic resources. Plant and Animal Genome XX Conference (January 14–18, 2012).

[58] Almeida CMVC de, Almeida CFG de (1987) Coleta de cacau silvestre no Estado de Rondônia, Brasil. Revista Theobroma 17: 65–92.

[59] Frankel OH, Brown AHD, Burdon J (1995b) The conservation of cultivated plants. In: The conservation of plant biodiversity. Cambridge, Great Britain: Cambridge University Press. 79–117.

[60] Laurent V, Risterucci AM, Lanaud C (1994) Genetic diversity in cocoa revealed by cDNA probes. Theoretical and Applied Genetics 88: 193–198. 24185926 10.1007/BF00225897

[61] Schroth G, Faria D, Araujo M, Bede L, Bael S, et al (2011) Conservation in tropical landscape mosaics: the case of the cacao landscape of southern Bahia, Brazil. Biodiversity and Conservation 20: 1635–1654.

[62] Fuller DQ, van Etten J, Manning K, Castillo C, Kingwell-Banham E, et al (2011) The contribution of rice agriculture and livestock pastoralism to prehistoric methane levels: An archaeological assessment. The Holocene 21: 743–759.

